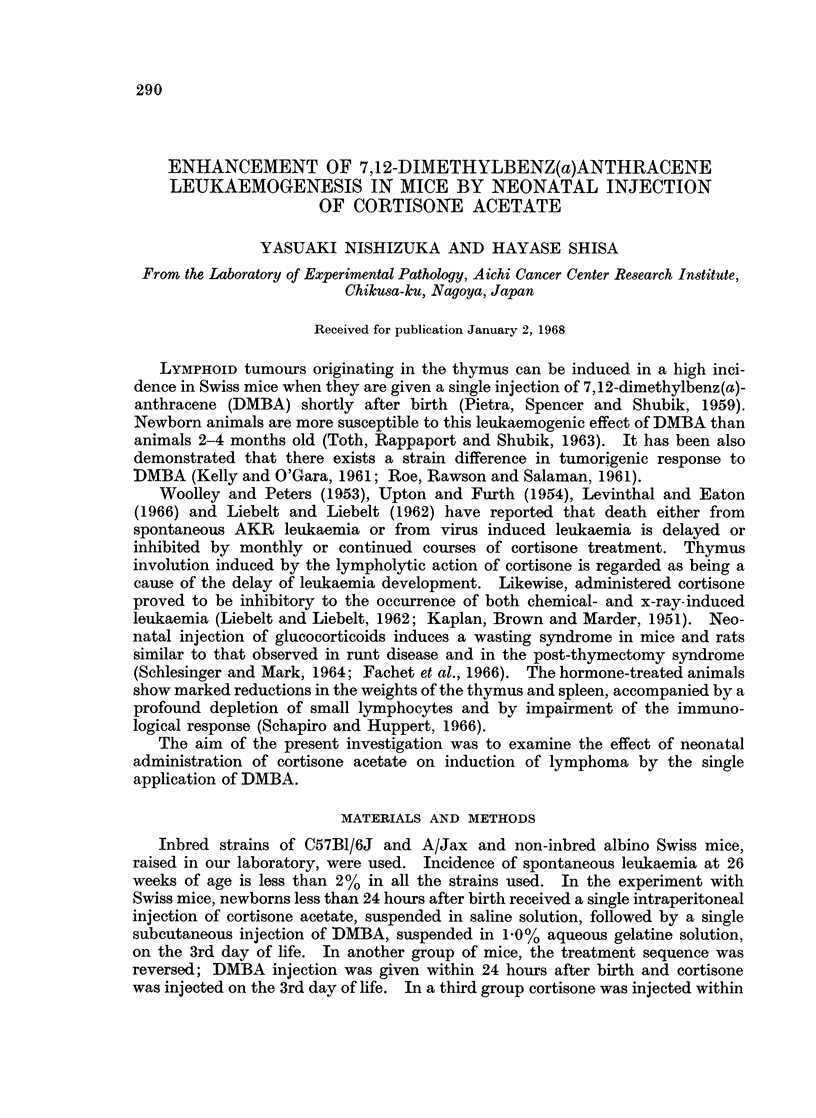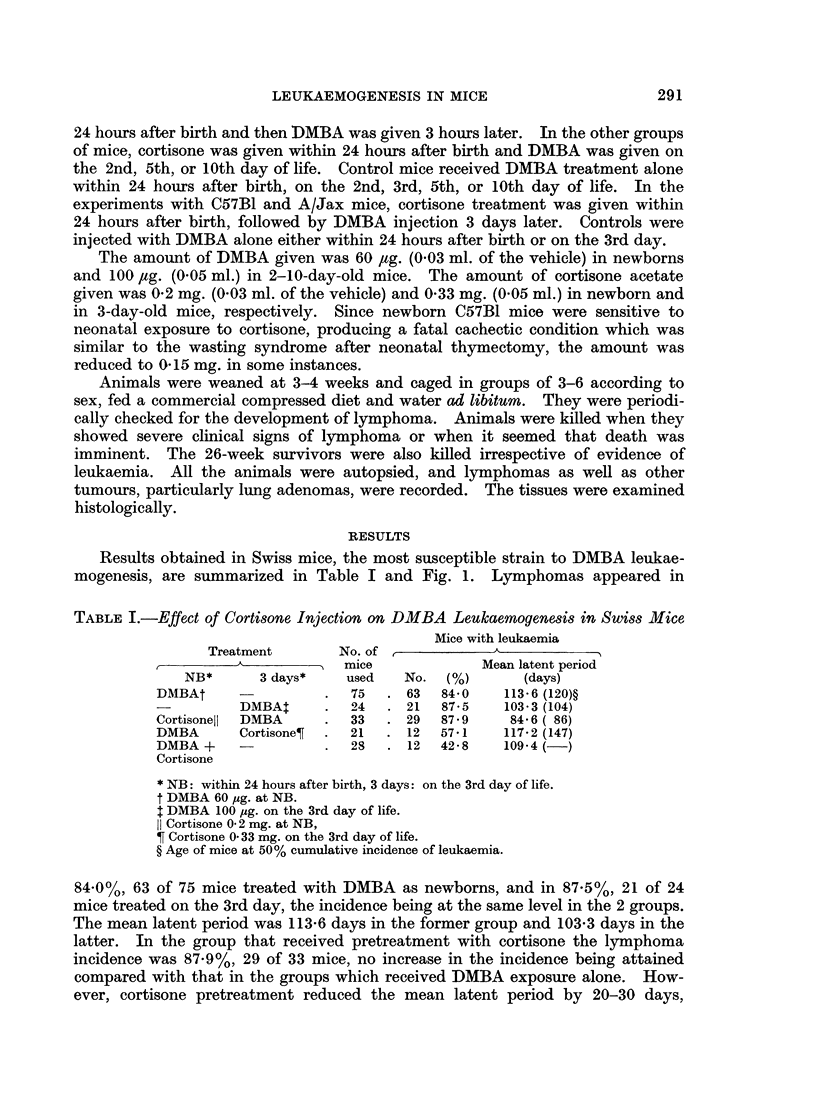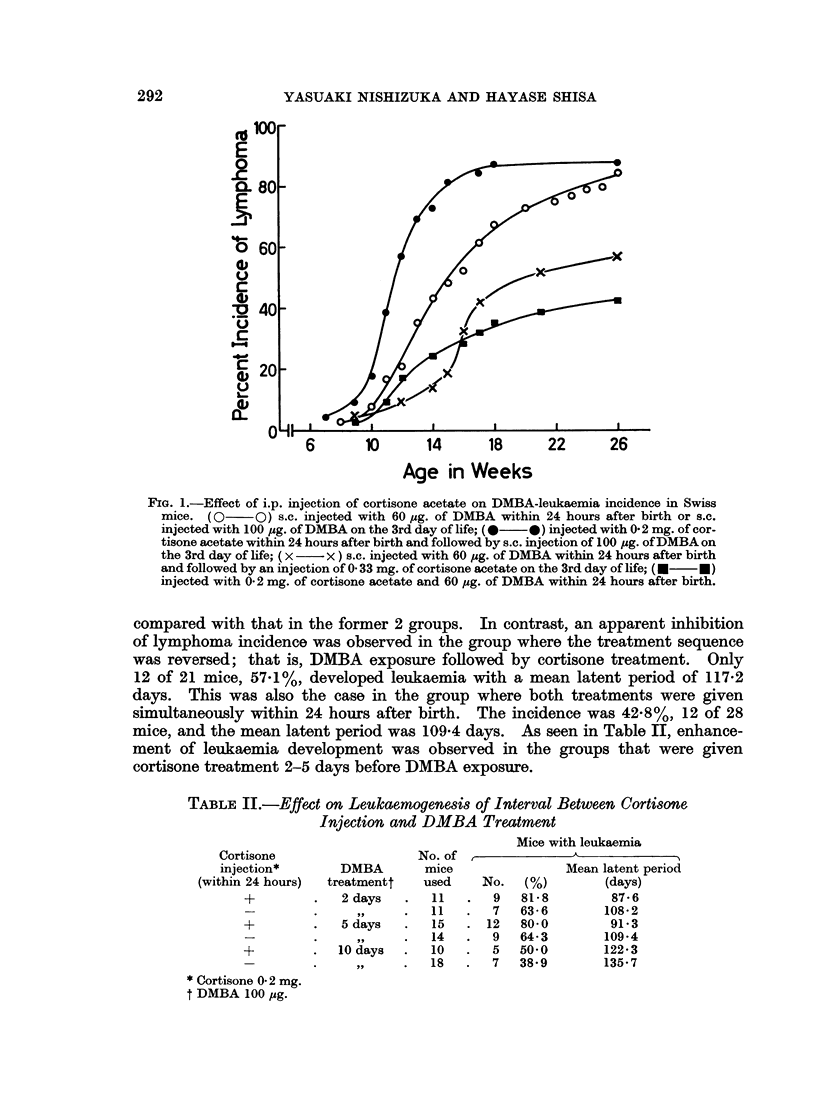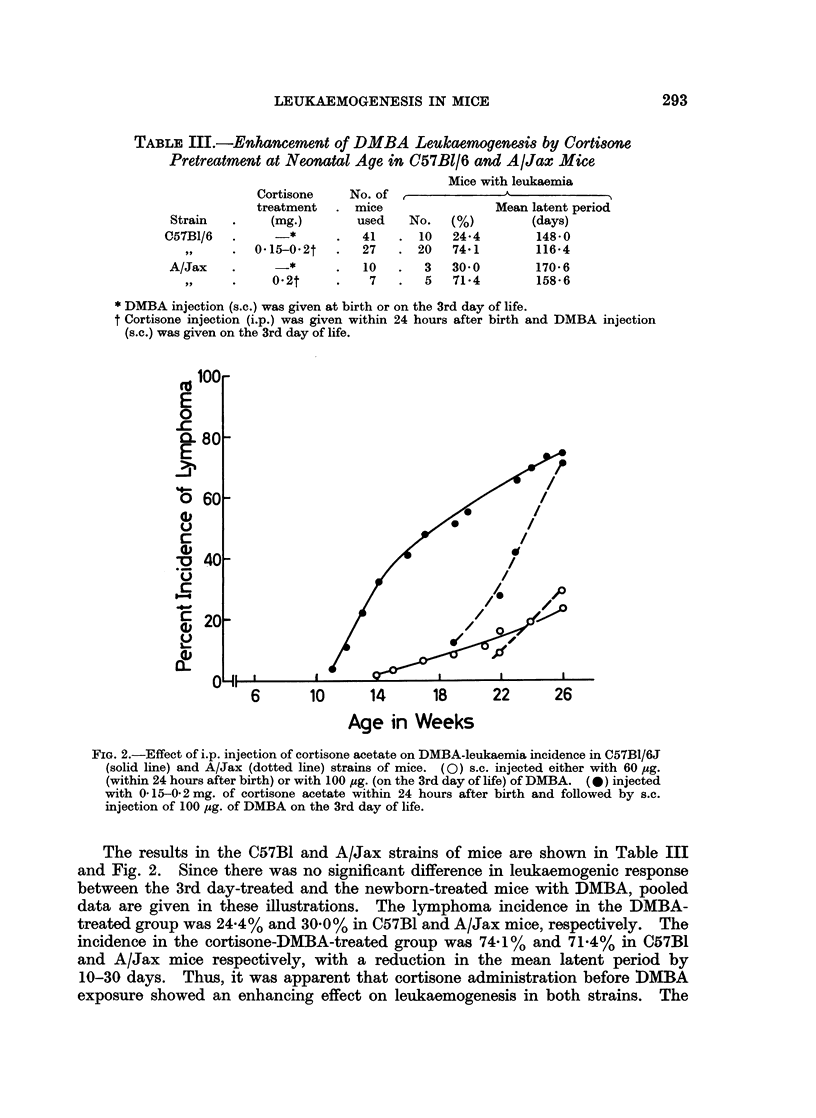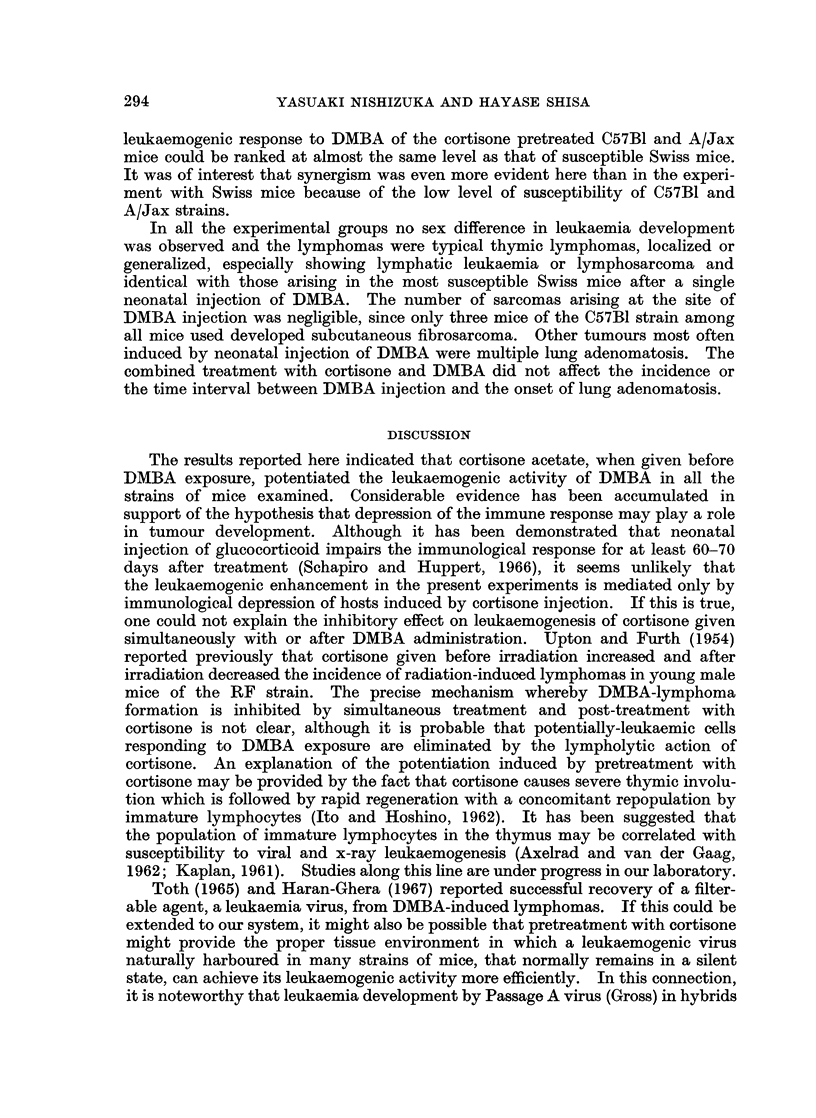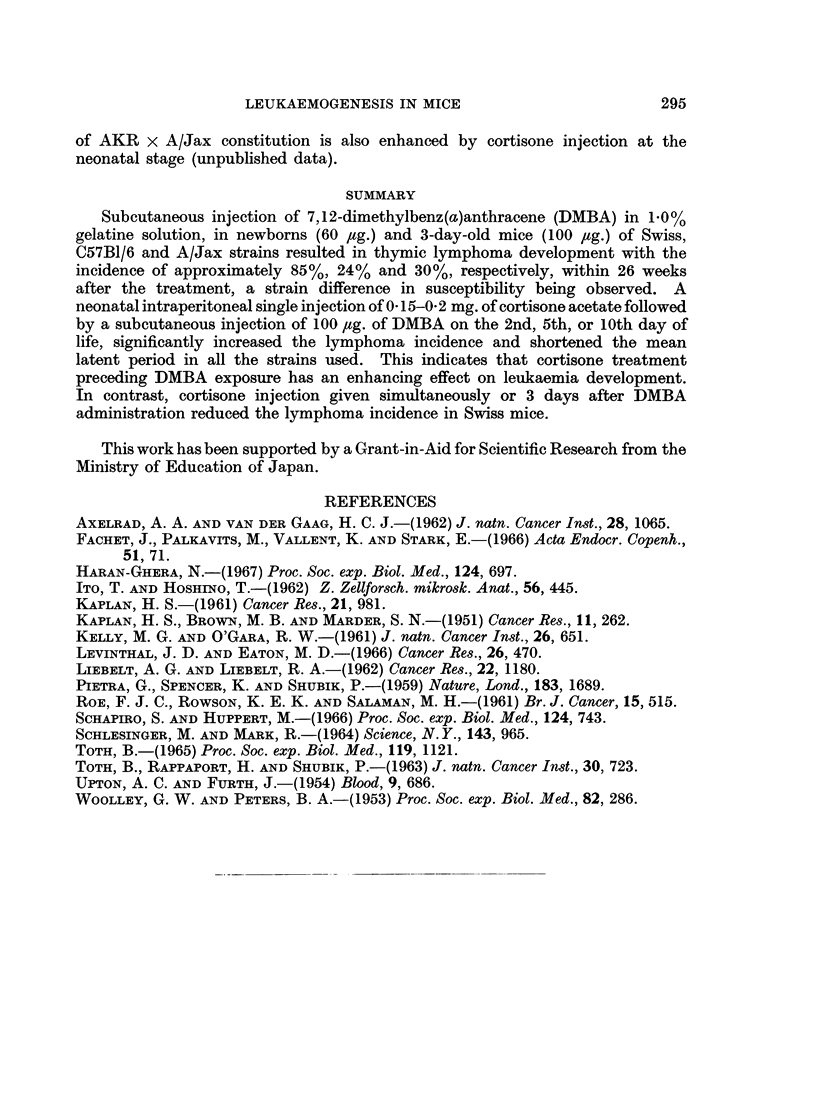# Enhancement of 7,12-dimethylbenz(a)anthracene leukaemogenesis in mice by neonatal injection of cortisone acetate.

**DOI:** 10.1038/bjc.1968.36

**Published:** 1968-06

**Authors:** Y. Nishizuka, H. Shisa


### ENHANCEMENT OF 7,12-DIMETHYLBENZ(*a*)ANTHRACENE LEUKAEMOGENESIS IN MICE BY NEONATAL INJECTION OF CORTISONE ACETATE

YASUAKI NISHIZUKA AND HAYASE SHISA

*From the Laboratory of Experimental Pathology, Aichi Cancer Center Research Institute, Chikusa-ku, Nagoya, Japan*

Received for publication January 2, 1968

LYMPHOID tumours originating in the thymus can be induced in a high incidence in Swiss mice when they are given a single injection of 7,12-dimethylbenz(*a*)-anthracene (DMBA) shortly after birth (Pietra, Spencer and Shubik, 1959). Newborn animals are more susceptible to this leukaemogenic effect of DMBA than animals 2–4 months old (Toth, Rappaport and Shubik, 1963). It has been also demonstrated that there exists a strain difference in tumorigenic response to DMBA (Kelly and O'Gara, 1961; Roe, Rawson and Salaman, 1961).

Woolley and Peters (1953), Upton and Furth (1954), Levinthal and Eaton (1966) and Liebelt and Liebelt (1962) have reported that death either from spontaneous AKR leukaemia or from virus induced leukaemia is delayed or inhibited by monthly or continued courses of cortisone treatment. Thymus involution induced by the lympholytic action of cortisone is regarded as being a cause of the delay of leukaemia development. Likewise, administered cortisone proved to be inhibitory to the occurrence of both chemical- and x-ray-induced leukaemia (Liebelt and Liebelt, 1962; Kaplan, Brown and Marder, 1951). Neonatal injection of glucocorticoids induces a wasting syndrome in mice and rats similar to that observed in runt disease and in the post-thymectomy syndrome (Schlesinger and Mark, 1964; Fachet *et al.*, 1966). The hormone-treated animals show marked reductions in the weights of the thymus and spleen, accompanied by a profound depletion of small lymphocytes and by impairment of the immunological response (Schapiro and Huppert, 1966).

The aim of the present investigation was to examine the effect of neonatal administration of cortisone acetate on induction of lymphoma by the single application of DMBA.

### MATERIALS AND METHODS

Inbred strains of C57Bl/6J and A/Jax and non-inbred albino Swiss mice, raised in our laboratory, were used. Incidence of spontaneous leukaemia at 26 weeks of age is less than 2% in all the strains used. In the experiment with Swiss mice, newborns less than 24 hours after birth received a single intraperitoneal injection of cortisone acetate, suspended in saline solution, followed by a single subcutaneous injection of DMBA, suspended in 1·0% aqueous gelatine solution, on the 3rd day of life. In another group of mice, the treatment sequence was reversed; DMBA injection was given within 24 hours after birth and cortisone was injected on the 3rd day of life. In a third group cortisone was injected within

24 hours after birth and then DMBA was given 3 hours later. In the other groups of mice, cortisone was given within 24 hours after birth and DMBA was given on the 2nd, 5th, or 10th day of life. Control mice received DMBA treatment alone within 24 hours after birth, on the 2nd, 3rd, 5th, or 10th day of life. In the experiments with C57Bl and A/Jax mice, cortisone treatment was given within 24 hours after birth, followed by DMBA injection 3 days later. Controls were injected with DMBA alone either within 24 hours after birth or on the 3rd day.

The amount of DMBA given was 60 μg. (0·03 ml. of the vehicle) in newborns and 100 μg. (0·05 ml.) in 2–10-day-old mice. The amount of cortisone acetate given was 0·2 mg. (0·03 ml. of the vehicle) and 0·33 mg. (0·05 ml.) in newborn and in 3-day-old mice, respectively. Since newborn C57Bl mice were sensitive to neonatal exposure to cortisone, producing a fatal cachectic condition which was similar to the wasting syndrome after neonatal thymectomy, the amount was reduced to 0·15 mg. in some instances.

Animals were weaned at 3–4 weeks and caged in groups of 3–6 according to sex, fed a commercial compressed diet and water *ad libitum*. They were periodically checked for the development of lymphoma. Animals were killed when they showed severe clinical signs of lymphoma or when it seemed that death was imminent. The 26-week survivors were also killed irrespective of evidence of leukaemia. All the animals were autopsied, and lymphomas as well as other tumours, particularly lung adenomas, were recorded. The tissues were examined histologically.

### RESULTS

Results obtained in Swiss mice, the most susceptible strain to DMBA leukaemogenesis, are summarized in Table I and Fig. 1. Lymphomas appeared in

TABLE I.—*Effect of Cortisone Injection on DMBA Leukaemogenesis in Swiss Mice*

| Treatment | | No. of mice used | Mice with leukaemia | | |
|---|---|---|---|---|---|
| NB* | 3 days* | | No. | (%) | Mean latent period (days) |
| DMBA† | — | 75 | 63 | 84·0 | 113·6 (120)§ |
| — | DMBA‡ | 24 | 21 | 87·5 | 103·3 (104) |
| Cortisone‖ | DMBA | 33 | 29 | 87·9 | 84·6 ( 86) |
| DMBA | Cortisone¶ | 21 | 12 | 57·1 | 117·2 (147) |
| DMBA + Cortisone | — | 28 | 12 | 42·8 | 109·4 (—) |

* NB: within 24 hours after birth, 3 days: on the 3rd day of life.
† DMBA 60 μg. at NB.
‡ DMBA 100 μg. on the 3rd day of life.
‖ Cortisone 0·2 mg. at NB,
¶ Cortisone 0·33 mg. on the 3rd day of life.
§ Age of mice at 50% cumulative incidence of leukaemia.

84·0%, 63 of 75 mice treated with DMBA as newborns, and in 87·5%, 21 of 24 mice treated on the 3rd day, the incidence being at the same level in the 2 groups. The mean latent period was 113·6 days in the former group and 103·3 days in the latter. In the group that received pretreatment with cortisone the lymphoma incidence was 87·9%, 29 of 33 mice, no increase in the incidence being attained compared with that in the groups which received DMBA exposure alone. However, cortisone pretreatment reduced the mean latent period by 20–30 days,

FIG. 1.—Effect of i.p. injection of cortisone acetate on DMBA-leukaemia incidence in Swiss mice. (○——○) s.c. injected with 60 μg. of DMBA within 24 hours after birth or s.c. injected with 100 μg. of DMBA on the 3rd day of life; (●——●) injected with 0·2 mg. of cortisone acetate within 24 hours after birth and followed by s.c. injection of 100 μg. of DMBA on the 3rd day of life; (×——×) s.c. injected with 60 μg. of DMBA within 24 hours after birth and followed by an injection of 0·33 mg. of cortisone acetate on the 3rd day of life; (■——■) injected with 0·2 mg. of cortisone acetate and 60 μg. of DMBA within 24 hours after birth.

compared with that in the former 2 groups. In contrast, an apparent inhibition of lymphoma incidence was observed in the group where the treatment sequence was reversed; that is, DMBA exposure followed by cortisone treatment. Only 12 of 21 mice, 57·1%, developed leukaemia with a mean latent period of 117·2 days. This was also the case in the group where both treatments were given simultaneously within 24 hours after birth. The incidence was 42·8%, 12 of 28 mice, and the mean latent period was 109·4 days. As seen in Table II, enhancement of leukaemia development was observed in the groups that were given cortisone treatment 2–5 days before DMBA exposure.

TABLE II.—*Effect on Leukaemogenesis of Interval Between Cortisone Injection and DMBA Treatment*

| Cortisone injection* (within 24 hours) | DMBA treatment† | No. of mice used | Mice with leukaemia | | |
|---|---|---|---|---|---|
| | | | No. | (%) | Mean latent period (days) |
| + | 2 days | 11 | 9 | 81·8 | 87·6 |
| − | ,, | 11 | 7 | 63·6 | 108·2 |
| + | 5 days | 15 | 12 | 80·0 | 91·3 |
| − | ,, | 14 | 9 | 64·3 | 109·4 |
| + | 10 days | 10 | 5 | 50·0 | 122·3 |
| − | ,, | 18 | 7 | 38·9 | 135·7 |

* Cortisone 0·2 mg.

† DMBA 100 μg.

TABLE III.—*Enhancement of DMBA Leukaemogenesis by Cortisone Pretreatment at Neonatal Age in C57Bl/6 and A/Jax Mice*

| Strain | Cortisone treatment (mg.) | No. of mice used | Mice with leukaemia No. | Mice with leukaemia (%) | Mice with leukaemia Mean latent period (days) |
|---|---|---|---|---|---|
| C57Bl/6 | —* | 41 | 10 | 24·4 | 148·0 |
| ,, | 0·15–0·2† | 27 | 20 | 74·1 | 116·4 |
| A/Jax | —* | 10 | 3 | 30·0 | 170·6 |
| ,, | 0·2† | 7 | 5 | 71·4 | 158·6 |

* DMBA injection (s.c.) was given at birth or on the 3rd day of life.
† Cortisone injection (i.p.) was given within 24 hours after birth and DMBA injection (s.c.) was given on the 3rd day of life.

FIG. 2.—Effect of i.p. injection of cortisone acetate on DMBA-leukaemia incidence in C57Bl/6J (solid line) and A/Jax (dotted line) strains of mice. (○) s.c. injected either with 60 μg. (within 24 hours after birth) or with 100 μg. (on the 3rd day of life) of DMBA. (●) injected with 0·15–0·2 mg. of cortisone acetate within 24 hours after birth and followed by s.c. injection of 100 μg. of DMBA on the 3rd day of life.

The results in the C57Bl and A/Jax strains of mice are shown in Table III and Fig. 2. Since there was no significant difference in leukaemogenic response between the 3rd day-treated and the newborn-treated mice with DMBA, pooled data are given in these illustrations. The lymphoma incidence in the DMBA-treated group was 24·4% and 30·0% in C57Bl and A/Jax mice, respectively. The incidence in the cortisone-DMBA-treated group was 74·1% and 71·4% in C57Bl and A/Jax mice respectively, with a reduction in the mean latent period by 10–30 days. Thus, it was apparent that cortisone administration before DMBA exposure showed an enhancing effect on leukaemogenesis in both strains. The leukaemogenic response to DMBA of the cortisone pretreated C57Bl and A/Jax mice could be ranked at almost the same level as that of susceptible Swiss mice. It was of interest that synergism was even more evident here than in the experiment with Swiss mice because of the low level of susceptibility of C57Bl and A/Jax strains.

In all the experimental groups no sex difference in leukaemia development was observed and the lymphomas were typical thymic lymphomas, localized or generalized, especially showing lymphatic leukaemia or lymphosarcoma and identical with those arising in the most susceptible Swiss mice after a single neonatal injection of DMBA. The number of sarcomas arising at the site of DMBA injection was negligible, since only three mice of the C57Bl strain among all mice used developed subcutaneous fibrosarcoma. Other tumours most often induced by neonatal injection of DMBA were multiple lung adenomatosis. The combined treatment with cortisone and DMBA did not affect the incidence or the time interval between DMBA injection and the onset of lung adenomatosis.

### DISCUSSION

The results reported here indicated that cortisone acetate, when given before DMBA exposure, potentiated the leukaemogenic activity of DMBA in all the strains of mice examined. Considerable evidence has been accumulated in support of the hypothesis that depression of the immune response may play a role in tumour development. Although it has been demonstrated that neonatal injection of glucocorticoid impairs the immunological response for at least 60–70 days after treatment (Schapiro and Huppert, 1966), it seems unlikely that the leukaemogenic enhancement in the present experiments is mediated only by immunological depression of hosts induced by cortisone injection. If this is true, one could not explain the inhibitory effect on leukaemogenesis of cortisone given simultaneously with or after DMBA administration. Upton and Furth (1954) reported previously that cortisone given before irradiation increased and after irradiation decreased the incidence of radiation-induced lymphomas in young male mice of the RF strain. The precise mechanism whereby DMBA-lymphoma formation is inhibited by simultaneous treatment and post-treatment with cortisone is not clear, although it is probable that potentially-leukaemic cells responding to DMBA exposure are eliminated by the lympholytic action of cortisone. An explanation of the potentiation induced by pretreatment with cortisone may be provided by the fact that cortisone causes severe thymic involution which is followed by rapid regeneration with a concomitant repopulation by immature lymphocytes (Ito and Hoshino, 1962). It has been suggested that the population of immature lymphocytes in the thymus may be correlated with susceptibility to viral and x-ray leukaemogenesis (Axelrad and van der Gaag, 1962; Kaplan, 1961). Studies along this line are under progress in our laboratory.

Toth (1965) and Haran-Ghera (1967) reported successful recovery of a filterable agent, a leukaemia virus, from DMBA-induced lymphomas. If this could be extended to our system, it might also be possible that pretreatment with cortisone might provide the proper tissue environment in which a leukaemogenic virus naturally harboured in many strains of mice, that normally remains in a silent state, can achieve its leukaemogenic activity more efficiently. In this connection, it is noteworthy that leukaemia development by Passage A virus (Gross) in hybrids of AKR × A/Jax constitution is also enhanced by cortisone injection at the neonatal stage (unpublished data).

SUMMARY

Subcutaneous injection of 7,12-dimethylbenz(*a*)anthracene (DMBA) in 1·0% gelatine solution, in newborns (60 $\mu$g.) and 3-day-old mice (100 $\mu$g.) of Swiss, C57Bl/6 and A/Jax strains resulted in thymic lymphoma development with the incidence of approximately 85%, 24% and 30%, respectively, within 26 weeks after the treatment, a strain difference in susceptibility being observed. A neonatal intraperitoneal single injection of 0·15–0·2 mg. of cortisone acetate followed by a subcutaneous injection of 100 $\mu$g. of DMBA on the 2nd, 5th, or 10th day of life, significantly increased the lymphoma incidence and shortened the mean latent period in all the strains used. This indicates that cortisone treatment preceding DMBA exposure has an enhancing effect on leukaemia development. In contrast, cortisone injection given simultaneously or 3 days after DMBA administration reduced the lymphoma incidence in Swiss mice.

This work has been supported by a Grant-in-Aid for Scientific Research from the Ministry of Education of Japan.

REFERENCES

Axelrad, A. A. and van der Gaag, H. C. J.—(1962) *J. natn. Cancer Inst.*, **28**, 1065.
Fachet, J., Palkavits, M., Vallent, K. and Stark, E.—(1966) *Acta Endocr. Copenh.*, **51**, 71.
Haran-Ghera, N.—(1967) *Proc. Soc. exp. Biol. Med.*, **124**, 697.
Ito, T. and Hoshino, T.—(1962) *Z. Zellforsch. mikrosk. Anat.*, **56**, 445.
Kaplan, H. S.—(1961) *Cancer Res.*, **21**, 981.
Kaplan, H. S., Brown, M. B. and Marder, S. N.—(1951) *Cancer Res.*, **11**, 262.
Kelly, M. G. and O'Gara, R. W.—(1961) *J. natn. Cancer Inst.*, **26**, 651.
Levinthal, J. D. and Eaton, M. D.—(1966) *Cancer Res.*, **26**, 470.
Liebelt, A. G. and Liebelt, R. A.—(1962) *Cancer Res.*, **22**, 1180.
Pietra, G., Spencer, K. and Shubik, P.—(1959) *Nature, Lond.*, **183**, 1689.
Roe, F. J. C., Rowson, K. E. K. and Salaman, M. H.—(1961) *Br. J. Cancer*, **15**, 515.
Schapiro, S. and Huppert, M.—(1966) *Proc. Soc. exp. Biol. Med.*, **124**, 743.
Schlesinger, M. and Mark, R.—(1964) *Science, N.Y.*, **143**, 965.
Toth, B.—(1965) *Proc. Soc. exp. Biol. Med.*, **119**, 1121.
Toth, B., Rappaport, H. and Shubik, P.—(1963) *J. natn. Cancer Inst.*, **30**, 723.
Upton, A. C. and Furth, J.—(1954) *Blood*, **9**, 686.
Woolley, G. W. and Peters, B. A.—(1953) *Proc. Soc. exp. Biol. Med.*, **82**, 286.